# Aspects épidémiologiques, nutritionnels et anatomopathologiques des cancers colorectaux dans la région du grand Casablanca

**DOI:** 10.11604/pamj.2019.32.56.10548

**Published:** 2019-01-31

**Authors:** Fatima Ezzahra Imad, Houda Drissi, Nezha Tawfiq, Karima Bendahhou, Nadia Tahiri Jouti, Abdellatif Benider, Driss Radallah

**Affiliations:** 1 Faculté des Sciences Ben M’Sik Université Hassan II, Casablanca, Maroc; 2 Centre Mohamed VI pour le Traitement des Cancers, CHU Ibn Rochd, Casablanca, Maroc; 3 Registre des Cancers de la Région du grand Casablanca, Maroc; 4 Faculté de Médecine et de Pharmacie, Université Hassan II de Casablanca, Maroc

**Keywords:** Cancer colorectal, épidémiologie, profil clinique, histologie, alimentation, Colorectal cancer, epidemiology, clinical profile, histology, diet

## Abstract

**Introduction:**

le cancer colorectal constitue un problème majeur de santé publique. L’objectif de notre étude est d’analyser le profil épidémiologique, nutritionnel, clinique et anatomo-pathologique des cancers colorectaux recrutés au CHU de Casablanca.

**Méthodes:**

notre étude cas-témoins a porté sur les patients pris en charge pour un cancer colorectal durant l’année 2015, comparés à des témoins non suivi pour un cancer.

**Résultats:**

l’âge moyen des patients était de 56,65 ans avec un écart type de 14,64. Le type histologique le plus fréquent chez nos patients était représenté par l’adénocarcinome Lieberkühnien avec une proportion de 82 %. L’analyse de l’indice de masse corporelle a permis de retrouver une obésité chez 50% des patients contre 20% des témoins et un diabète chez 19% des patients versus 8% des témoins (p < 0,019). Par ailleurs, l’étude du régime alimentaire des patients comparé à celui des témoins semble montrer que la moyenne de la fréquence de consommation hebdomadaire de viandes rouges est plus élevée chez les patients que chez les témoins (4,24 vs 3,26; p = 0,009) et inversement pour la consommation du poisson (0,97 contre 1,76; p = 0,0001). En revanche, la moyenne de consommation des légumes et des fruits est plus faible chez les patients que chez les témoins (5,00 vs 9,50; p = 0,0001). Concernant les habitudes toxiques de nos patients, 32% des patients étaient fumeurs vs 13 % des témoins.

**Conclusion:**

nos résultats montrent que la prise de conscience à propos du régime alimentaire et des changements dans nos habitudes de vie pourrait réduire l’incidence du cancer colorectal et par conséquent la mortalité et la morbidité.

## Abstract (en)

**Introduction:**

colorectal cancer is a major public health problem. This study aims to analyze the epidemiological, nutritional, clinical and anatomopathological features of patients with colorectal cancer at the University Hospital in Casablanca.

**Methods:**

our case-control study focused on patients assigned to treatment for colorectal cancer in 2015 compared with a control group that didn’t have cancer.

**Results:**

the average age of our patients was 56.65 years, with a standard deviation of 14.64. The most common histological type of cancer in study patients was Lieberkhünien adenocarcinoma with a proportion of 82%. The analysis of the body mass index showed that 50% of study patients were obese vs 20% of patients in the control group and that 19% of study patients had diabetes vs 8% patients in the control group (p< 0.019). Moreover, the analysis of dietary habits in study patients compared to those of patients in the control group showed that the average frequency of weekly consumption of red meat was higher in study patients than in the control group (4.24 vs 3.26; p = 0.009) and , conversely, for the consumption of fish (0.97 vs 1.76; p = 0.0001). On the other hand, the average consumption of vegetables and fruits was lower in study patients than in the control group (5.00 vs 9.50; p = 0.0001). Concerning the toxic habits of our patients, 32% of study patients were smokers vs. 13% of patients in the control group.

**Conclusion:**

our results show that awareness about our dietary habits and changes in our living habits could reduce the incidence of colorectal cancer and therefore mortality and morbidity.

## Introduction

Le cancer colorectal, par sa fréquence et sa gravité, représente un sérieux problème de santé publique dans le monde. Il occupe la 3^ème^ place par son incidence, six cent quatre-vingt-quatorze mille décès par cancer colorectal sont enregistrés chaque année dans le monde [1]. Au Maroc, ce type de cancer occupe la 3^ème^ place chez l’homme et la 4^ème^ place chez la femme selon le dernier registre des cancers de la région du grand Casablanca [2]. Sa gravité est liée essentiellement au retard diagnostic. Sur le plan pronostic, la survie est liée au stade. Après traitement locorégional, un malade sur deux développe des métastases. Quatre-vingt à quatre-vingt-dix pour cent de ces patients décèdent de leurs métastases. L’objectif de l’étude est de déterminer dans notre contexte marocain, les facteurs de risque du cancer colorectal, notamment le lien de cause à effet avec le type d’alimentation. Le but ultime est de pouvoir élaborer des recommandations en matière de prévention des risques afin de mettre en œuvre une politique de prévention primaire.

## Méthodes

### Population d’étude

Il s’agit d’une étude descriptive menée au centre Mohammed VI pour le traitement des cancers de Casablanca. Nous avons inclus tous les cas de cancer colorectal de manière consécutive nouvellement diagnostiqué et confirmé par un examen histologique durant une période de 12 mois allant du 1^er^ janvier au 31 décembre 2015, et des témoins indemnes de toute maladie cancéreuse (patients parmi les admis au Centre des Consultations de Dermatologie et d’Ophtalmologie de CHU Ibn Rochd Casablanca). Après accord du comité d’éthique local et consentement éclairé signé par le patient et le témoin après avoir reçu l’information nécessaire à sa prise de décision. Les patients et les témoins ont été appariés selon l’âge et le sexe. Le recueil des données s’est fait de manière prospective à partir des dossiers de patients et au moyen d’un questionnaire standardisé englobant plusieurs informations : données sociodémographiques, antécédents personnels et familiaux de cancer, mode de vie, tabagisme, données cliniques et anatomopathologiques (localisation du cancer, histologie, classification TNM, stade et grade de différenciation…), hygiène de vie et habitudes alimentaires. Ce dernier volet qui regroupe la nature, la fréquence, la diversité et la quantité des aliments consommés est adapté au contexte marocain en se basant sur l’étude European Prospective Investigation into Cancer and Nutrition (Epic).

### Critères d’inclusion

Dans l’étude, sont inclus: 1) tous les patients présentant un cancer du côlon ou rectum avéré, confirmé par l´histologie; 2) patients n´ayant pas des troubles cognitifs. Les critères d’exclusion étaient: 1) tumeur non confirmée par l´histologie; 2) patients ayant des troubles cognitifs. La saisie des données a été réalisée par Microsoft office Excel (2016) et la gestion et l’analyse statistique des données épidémiologiques ont été faites à l’aide du logiciel R. L’étude association en croisant les variables entre les différents groupes a été évaluée par le test de Chi^2^. Le seuil de significativité retenu est p < 0,05.

## Résultats

Durant notre période d’étude, On a pu recruter 100 cas de cancer colorectal et 100 témoins.

### Profil épidémiologique

**Sexe:** selon nos résultats, il existe une légère prédominance masculine avec 54% d’hommes et 46% de femmes et un sex-ratio de 1,17 (Tableau 1).

**Tableau 1:** caractéristiques sociodémographiques de la population d’étude

	Patients N=100	Témoins N=100
**Age moyen (±ecart type)**	**56,65 ± 14,64**	
**Sexe**		
Femme	46	46
Homme	54	54
**Statut matrimonial**		
Célibataire	10	14
**Marié (e)**	81	72
Divorcé (e)	2	3
Veuf (ve)	7	11
**Milieu de résidence**		
Urbain	75	83
Suburbain	6	2
Rural	18	6
**Niveau d'étude**		
Analphabète	44	20
Ecole coranique	8	7
Primaire	25	15
Secondaire	14	27
Supérieur	9	31

**Âge:** l’âge moyen dans notre étude au moment du diagnostic était de 56,65±14,64. Le risque du cancer colorectal augmentait avec l’âge quel que soit le sexe. La tranche d’âge la plus touchée était comprise entre 60-65 ans.

**Statut matrimonial:** la répartition des patients selon le statut matrimonial rapportait que 81% des patients vs 72% des témoins étaient mariés.

**Milieu de résidence:** dans la population étudiée, 80% résidait dans un milieu urbain.

**Niveau d’étude:** cinquante pour cent des patients étaient analphabètes.

**Diabète:** le diabète associé au cancer colorectal a été retrouvé chez 19% des patients contre 8% chez les témoins (P = 0,02).

**Antécédents familiaux de cancer colorectaux et d´autres types de cancer:** en se basant sur les données du questionnaire, les antécédents familiaux de cancer colorectal ont été retrouvés chez 7% des patients. Les antécédents familiaux d’autres types de cancer ont été notés chez 16% des patients contre seulement 7% des témoins (Tableau 2). Concernant les antécédents familiaux de cancers rentrant dans le spectre du syndrome de Lynch, les cancers du sein, des poumons, du foie, de l’estomac, du pancréas et de l’ovaire étaient présents chez 42%,15%, 12%, 12%, 4% et 4%, respectivement.

**Tableau 2:** répartition de la population d’étude selon le mode de vie et les antécédents familiaux et médicaux de cancer

	Patients N=100 (%)	Témoins N=100 (%)	P
**ATCD Familiaux de CCR**			**P = 0,08**
Oui	7	2	
Non	93	98	
**ATCD Familiaux d'autres types de cancer**			**P= 0,03**
Oui	16	7	
Non	84	93	
**Tabagisme**			**P=0,001**
Non- fumeur	68	87	
fumeur	32	13	
**Contraception orale**			**P = 0,3**
Oui	18	13	
Non	82	87	
**Diabète**			**P< 0,02**
Oui	19	8	
Non	81	92	
**Aspirine**			**P = 0,002**
Oui	4	17	
Non	96	83	
**IMC**			**P=0,0001**
Insuffisance pondérale (IMC<18.5)	1	11	
Poids normal (18.5<IMC<24)	24	56	
Surpoids (25< IMC<29.9)	53	26	
Obésité (IMC >30)	14	6	
**Activité physique**			
Travail de forte intensité	41	25	**P=0,01**
Travail d’intensité modéré	61	74	**P=0 ,03**
Marche	81	88	**P=0,12**
Sport fort	3	12	**P=0,01**
Sport modéré	6	29	**P=0,0001**

**Utilisation de contraceptifs oraux:** la prise de contraceptifs oraux a été retrouvée chez 18% des patientes et 13% des témoins (P = 0,3).

**Aspirine:** la prise d’aspirine a été retrouvée chez 4% des patients, alors qu’elle s’élève à 17% chez les témoins (P = 0,002).

**Habitudes toxiques:** dans notre série, les patients tabagiques représentaient 32% des cas, contre 13% des témoins. Ces résultats indiquent une association significative entre le tabagisme et la survenue de cancer colorectal (P = 0,001).

**Indice de masse corporelle:** l’analyse de l’indice de masse corporelle a permis de retrouver un surpoids chez 53% des patients contre 26% des témoins et une obésité chez 14% des patients *vs* 6% des témoins (P = 0,0001).

**Activité physique:** dans notre étude, la pratique d’une activité sportive de moyenne intensité ou de forte intensité était plus faible chez les patients, avec 3% des cas *vs* 12% des témoins (P = 0,01) et 6% des cas *vs* 29% des témoins (P = 0,0001), respectivement. Paradoxalement, le métier où prédomine le travail physique de forte intensité est corrélé à un risque plus élevé de cancer colorectal, ce métier est retrouvé chez 41% des patients contre 25% des témoins (p = 0,01).

### Habitudes alimentaires

**La consommation de boissons (café, thé et soda):** la consommation moyenne de café était plus faible chez les patients que chez les témoins (1,15 *vs* 3,30). Cependant, pour les autres boissons (soda, alcool et thé) la consommation est plus élevée chez les malades par rapport aux témoins avec 1,28 *vs* 0,53, 1,69 *vs* 1,00 et 18,99 *vs* 16,18, respectivement. Il existe ainsi une association significative entre la consommation de ces boissons et la survenue du CCR (Tableau 3).

**Tableau 3:** consommation alimentaire de la population d'étude

Consommation moyenne par semaine			
	Patients N=100	Témoins N=100	P
**Boissons**			
Lait	1,11	1,42	**P=0,3**
Café au lait	2,24	2,94	**P=0,2**
Café	1,15	3,30	**P=0,0001**
Thé	18,99	16,18	**P=0,02**
Soda	1,28	0,53	**P=0,003**
Alcool	1,69	1,00	**P=0,7**
**Viandes**			
Viandes rouges	4,24	3,26	**P=0,009**
Volailles	3,34	3,55	**P=0,5**
Poissons	0,97	1,76	**P=0,0001**
Saucisses	0,14	0,33	**P=0,04**
Charcuteries	1,16	0,39	**P=0,003**
**Légumes**			
Légumes frais	0,18	0,72	**P=0,001**
Légumes cuits	4,83	6,27	**P=0,006**
Légumes secs	0,78	0,73	**P=0,8**
Pomme de terre	5,46	2,05	**P=0,001**
**Fruits**			
Fruits	1,92	4,69	**P=0,0001**

Chaque valeur représente la moyenne, la comparaison des moyennes entre les patients et les témoins est effectué par le test t de student après analyse de la variance *P<*0.05

**La consommation de viandes rouges:** la consommation moyenne de viandes rouges était plus élevée chez les patients que chez les témoins (4,24 *vs* 3,26; p = 0,009, Tableau 3). La consommation de viandes rouges montre une corrélation positive avec la survenue du cancer colorectal (CCR).

**La consommation de légumes:** la consommation moyenne de légumes crus était de 0,18 chez les patients et 0,72 chez les témoins. Pour les légumes cuits, elle était de 4,83 chez les patients et de 6,27 chez les témoins. Il existe une association significative entre la consommation faible des légumes et la survenue de la maladie.

**La consommation de fruits:** la consommation moyenne de fruits frais était plus faible chez les patients que chez les témoins (1,92 *vs* 4,69; p = 0,0001). Cette consommation montre une association significative avec la survenue du CCR chez nos patients.

### Profil anatomoclinique

Le cancer colorectal touchait, chez notre population d’étude, le côlon dans 52% des cas et le rectum dans 48%. Le cancer colique a été retrouvé principalement sur le colon sigmoïde chez 33% de nos patients. D’après les résultats anatomo-pathologiques présentés, nous avons noté une prédominance de l’adénocarcinome *lieberkühnien* (82%) par rapport aux autres types de cancers (carcinomes mucineux et SAI), dont 60% sont de type moyennement différencié. Les emboles vasculaires étaient retrouvées dans 28% des cas et les engainements péri-nerveux dans 14% des cas (Tableau 4). L’examen anatomopathologique a permis de déterminer le stade de la tumeur selon la classification TNM [3] (UICC 2010). Quarante pour cent de la population étudiée avait un cancer colorectal au stade III. Le stade IV a été retrouvé chez 36% des patients avec des métastases intéressant principalement le foie et les poumons. Le CCR se présentait essentiellement sous forme ulcéro-bourgeonnante dans 62% des cas. Seize pour cent des patients avaient des polypes associés à la tumeur (Tableau 5).

**Tableau 4:** caractéristiques histologiques des CCR

	N=100 (%)
Type histologique	
ADK Liberkhunien	82
ADK Mucineux	15
SAI	1
Autres	2
**Degré de différenciation**	
Bien différencié	27
Moyennement différencié	60
Peu différencié	12
Indifférencié	1
**Emboles vasculaires**	
Absents	72
Présents	28
**Engainements périnerveux**	
Absents	86
Présents	14

**Tableau 5:** caractéristiques endoscopiques des CCR

	N =100 (%)
**Siège de la tumeur**	
Rectum	48
Colon sigmoïde	33
Colon gauche	11
Colon transverse	9
Colon droit	16
**Aspect**	
Ulcéro-bourgeonnant	62
Infiltrant	36
Bourgeonnant	2
**Polypes associés**	
Absents	84
Présents	16
**Métastases**	
Absents	64
Présents	36
**localisations de métastases**	
Foie	20
Poumon	4
Autres	4

## Discussion

Notre étude cas-témoin a mis en relief plusieurs aspects reliant les quatre grands types de facteurs de risque à savoir: héréditaires, hormonaux, nutritionnels et environnementaux. Dans notre série, le faible niveau socio-économique lié au niveau d’instruction bas (50% sont des analphabètes) sont des facteurs favorisants le cancer colorectal dans la population étudiée. L’âge moyen de nos patients atteints de cancer colorectal est de 56,65±14,64 ans. Ce résultat est comparable à celui notifié (53,5 ans) dans le registre des cancers de Rabat [4] et également celui relevé en 2014 (54 ans) par El Housse *et al.* [5] mais très en deçà de celui (70,2 ans) noté dans une série française [6]. La répartition du cancer colorectal selon le sexe montre un sex-ratio équilibré, avec une légère prédominance masculine. Cette prédominance masculine a été notée auparavant dans une étude nationale du CHU Hassan II de Fès [7].

Par ailleurs, une association entre diabète et cancer colorectal a clairement été démontrée. Dans notre étude, nous avons observé chez les patients diabétiques une augmentation de survenue de CCR. Nos résultats sont comparables à beaucoup de recherches qui ont focalisé l’attention sur la relation entre diabète et CCR. En effet, neuf études de cohorte et 6 études cas-témoin ont expliqué ce risque par l’hyperinsulinémie mais également par un temps de transit intestinal plus long entraînant un plus grand temps d’exposition aux agents potentiellement cancérigènes pour la muqueuse intestinale [8]. La présence d’antécédents familiaux de cancer colorectal est un facteur de risque important d’apparition du cancer colorectal. Dans notre série, 7% des patients avaient au moins un antécédent familial de cancer colorectal. Ce chiffre concorde avec ceux retrouvés dans la littérature. Une méta-analyse incluant 59 études a estimé que le risque relatif de développer un CCR en cas d’antécédent familial au premier degré est de 2,24. Ce risque passait à 3,97 si deux antécédents familiaux au premier degré existaient [9].

D’un autre côté, il est avéré que l’aspirine confère une protection contre le CCR. Une analyse de cohorte de 662 hommes et 424 femmes inscrits dans le Cancer Prevention Study (CPS) aux États-Unis a montré que l’utilisation d´aspirine au moins 16 fois par mois a été associée avec un risque réduit de 40% de la mortalité par cancer du côlon sur une période de 5 ans [10]. Cet effet protecteur de l’aspirine est visible dans nos résultats. Cependant d’autres études de cohorte et cas-témoins ont observé une association inverse entre l’utilisation d’aspirine et le risque de CCR [11].

En outre, il a été estimé que 12% des CCR sont imputables au tabagisme. Sa consommation favorise une apparition précoce de CCR et engendre des mutations au niveau de l’ADN de la muqueuse digestive [12]. Nos résultats sont en faveur d’une association entre tabagisme et cancer colorectal et concordent avec les trois méta-analyses ayant constaté que les fumeurs actuels présentent un risque accru de cancer colorectal que les non-fumeurs [13].

Par ailleurs, nos résultats dévoilent que l’alcool est un facteur favorisant le cancer colorectal chez nos patients. Le principal métabolite de l’alcool dans le colon, l’acétaldéhyde a récemment été classé par l’OMS comme carcinogène du groupe 1. Les études épidémiologiques ont généralement rapporté des associations positives entre le cancer colorectal et l’alcool [14]. Une méta-analyse réalisée à partir de 8 cohortes nord-américaines et européennes a montré qu’au-delà de 45 g d’alcool/j, le risque relatif de cancer colorectal était de 1,41 (IC = 1,16-1,72) [15]. L’analyse de l’indice de masse corporelle a permis de retrouver un surpoids chez 53% des patients contre 26% des témoins et une obésité chez 14% des patients VS 6% des témoins. Le surpoids et l’obésité ont déjà été associés de façon convaincante avec le risque de cancer colorectal En effet, l’excès calorique et la sédentarité pourraient agir comme facteurs promoteurs de la cancérogénèse par le biais de l’hyperinsulinisme et/ou de résistance à l’insuline. L’insuline aurait un rôle stimulant sur les récepteurs à l’insulin-like growth-factors (IGF1) des cellules coliques en phase d’initiation [16].

Le cancer colorectal représente le cancer sur lequel il existe le plus d’évidence sur l’effet bénéfique de l’activité physique. Dans notre étude, la pratique d’activité sportive était plus faible chez les patients que les témoins, elle semble être corrélée à une diminution du risque de cancer colorectal. Ce résultat est en accord avec une récente étude cohorte européenne qui a porté sur 347 hommes et 237 femmes [17]. Les effets bénéfiques de l’activité physique ont été démontrés essentiellement sur la diminution de l’insulinorésistance et sur la prise de poids (et donc de masse grasse). Un mécanisme à effet local a été également proposé pour expliquer les effets protecteurs de la pratique d’une activité physique régulière sur la survenue de ce cancer, et l’augmentation de la motilité intestinale. En effet, l’activité physique induit une réduction du temps de transit gastro-intestinal et donc une diminution de l’opportunité pour les cancérigènes d’être en contact avec la muqueuse colique [18].

Par ailleurs, l’alimentation contribue grandement à l’augmentation du taux d’incidence du cancer colorectal dans le monde. On estime qu’elle pourrait expliquer jusqu’à 80% des différences d’incidence de cancer observées entre les pays [19]. Le principal volet de cette étude concerne l’exploration du statut nutritionnel dans l’étiologie du cancer colorectal. Une attention particulière est orientée vers la consommation de viandes rouges qui est significativement élevée chez nos patients comparée aux témoins. Ces résultats sont concordants avec plusieurs études épidémiologiques ayant investigué le rôle éventuel de la consommation de viande dans la survenue de cancer colorectal. L’étude de cohorte européenne EPIC montre que les cas de cancers colorectaux présentant une mutation du gène Apc provenaient des personnes consommant le plus de viandes rouges et de charcuteries [20]. Les agents pro-cancer de la viande rouge pourraient être également l’excès de fer héminique, les mutagènes induits par la cuisson ou l’excès de graisses. A ces agents, qui sont aussi présents dans les charcuteries, s’ajoute le nitrite utilisé comme additif. Quant à La viande blanche qui ne contient que peu de fer héminique, elle n’est pas associée à la cancérogenèse colorectale contrairement à la viande rouge [21]. Il est aussi à noter que, d’après notre étude et selon l’étude européenne EPIC, il semble également que le poisson soit un facteur protecteur, la consommation de poisson était inversement et significativement associée au risque de cancer colorectal. Il n’y a pas de mécanisme formellement démontré pour expliquer l’effet protecteur du poisson. Des études chez les animaux et *in vitro* indiquent que les acides gras à chaîne longue caractéristiques de l’huile de poisson pourraient inhiber la cancérogenèse [22]. Sans compter que le poisson est une des rares sources alimentaires de vitamine D, une vitamine connue pour réduire le risque de cancer colorectal.

Beaucoup de recherches ont focalisé l’attention sur la consommation des légumes. La méta-analyse des données des études de cohorte a montré une diminution de risque de CCR de 10% pour une consommation de l0g/jour [21]. Dans notre étude, les patients sont moins consommateurs de légumes que les témoins. Nos résultats suggèrent un effet protecteur des légumes crus en rapport avec leur richesse en fibres. Murphy *et al.* ont montré qu’un régime riche en fibres est inversement associé au risque de CCR et pourrait jouer un rôle préventif contre cette pathologie [23]. Ces fibres seraient protectrices en accélérant le transit digestif, en diluant le contenu colique et en diminuant ainsi le temps de contact des produits mutagènes avec la muqueuse colique. De plus, les résultats de notre étude montrent une association significative entre la consommation des fruits et la réduction du risque du CCR. Nos résultats concordent avec plusieurs études [21] qui sont en faveur d’un effet protecteur des fruits. Plusieurs composants des fruits peuvent expliquer cet effet protecteur, ils sont riches en vitamines antioxydantes, en fibres, en flavonoïdes et caroténoïdes.

En revanche, dans notre étude, les patients sont moins consommateurs de café que les témoins. Ce résultat est en accord avec une étude menée par Rashmi *et al.* ayant mis la lumière sur un effet favorable du café sur le risque de cancer colorectal. Les auteurs ont suggéré une réduction de 39% du risque de cancer colorectal, pour les gros consommateurs de café, soit 6 tasses par jour ou plus [24]. Sur le plan histologique, il ressort de l’analyse des données anatomopathologiques des patients que l’adénocarcinome *Lieberkühnien* est le type le plus fréquent (82%), touchant avec prédilection le côlon dans 52% des cas et le rectum dans 48%. Le cancer colique a été retrouvé principalement sur le colon sigmoïde chez 33% de nos patients. Ce résultat est comparable à ceux relevés à Rabat [4], à Marrakech [25], ainsi qu’à Casablanca [2]. Néanmoins, la répartition topographique du cancer du côlon dans notre série est différente de celle retrouvée dans une population française, avec 49,4% de localisation droite suivie du côlon sigmoïde [6]. En outre, il a été constaté depuis les années 80, un glissement progressif des cancers du côlon gauche et du rectum au profit du côlon droit en Europe et aux États-Unis d’Amérique. La localisation sur le côlon proximal tend à devenir plus fréquente que la localisation sur le côlon distal lorsque le niveau de développement socioéconomique d’un pays s’élève [26].

## Conclusion

Au Maroc, l’incidence du cancer colorectal est à l’heure actuelle en augmentation. Nous assistons à une véritable transition épidémiologique avec des changements dans le style de vie, le passage d’une alimentation à base de céréales et de légumineuses à une alimentation riche en viandes, et une consommation de plus en plus fréquente des « fast-food » et des produits industrialisés. Ces transformations dans notre mode vie font craindre une augmentation de l’incidence des cancers colorectaux dans notre pays. Ainsi et à la lumière de nos résultats, des recommandations peuvent être suggérées dans le cadre de la prévention primaire. Il est possible d'éviter le cancer colorectal d’une part en adoptant une hygiène de vie appropriée avec une alimentation saine diversifiée et équilibrée (peu de viande rouge, de charcuterie et de grillades, mais plutôt du poisson, des céréales complètes, des fruits et des légumes), la pratique régulière d'exercice physique, l’arrêt du tabac et de l’alcool et d’autre part en évitant la consommation excessive d'aliments transformés, surchargés de sucres et de gras. Des études plus ciblées doivent être conduites pour établir l’association entre ces facteurs de risque et la survenue du cancer colorectal chez la population marocaine.

### Etat des connaissances actuelles sur le sujet

- Le cancer colorectal, par sa fréquence et sa gravité, représente un sérieux problème de santé publique dans le monde. Au Maroc, l’incidence de ce cancer est faible par rapport aux pays développés - Néanmoins, cette incidence est en constante croissance comme le prouve l’évolution de l’incidence de ce cancer dans la région du grand Casablanca qui est passée de 7,3 cas/100000 habitants en 2004-2007 à 9,6 cas/1.000.000 habitants en 2008-2012 pour les deux sexes confondus - Il est devenu le premier cancer digestif, surclassant le cancer de l’estomac;
- La curabilité du cancer colorectal est en amélioration, grâce aux progrès thérapeutiques, mais des défis restent à relever en rapport avec les quatre grands types de facteurs de risque à savoir: héréditaires, hormonaux, nutritionnels et environnementaux.

### Contribution de notre étude à la connaissance

- Ce travail est réalisé pour la première fois au centre Mohammed VI pour le traitement des cancers l’un des deux plus grands centre pour la prise en charge et le traitement des cancers au Maroc - Notre étude cas-témoin a mis en relief plusieurs aspects reliant les quatre grands types de facteurs de risque à savoir: héréditaires, hormonaux, nutritionnels et environnementaux;
- Cette étude a permis de mettre en évidence l’âge de survenue du cancer colorectal de 10 ans plus jeune que l’âge rapporté dans la littérature occidentale;
- Notre étude a démontré le lien entre certains facteurs de risque connus dans la littérature et le cancer colorectal dans notre contexte: le niveau d’études, les antécédents familiaux de cancer, l’activité physique, l’obésité, l’intoxication alcoolo-tabagique, l’utilisation de l’aspirine - L’association était moins évidente pour le diabète, la contraception orale, tandis que notre population étudiée est sujette à un déséquilibre alimentaire qui est en faveur du développement ou de la complication de cancer colorectal, d’où la nécessité d’adopter un régime alimentaire bien codifié.

## Conflits d’intérêts

Les auteurs ne déclarent aucun conflit d’intérêts.
